# Home Again

**Published:** 1882-04

**Authors:** 


### HOME AGAIN.

Dr. C. M. Wright, who has spent the last ten years in dental practice in Basle, Switzerland, has returned to Cincinnati, his former home, and has taken up practice, and designs to make this his field of future work. The Doctor, while abroad, was eminently successful, enjoying a lucrative practice and establishing a reputation that any one might envy. It is presumed that the Doctor has his exchequer in a very comfortable shape; and there is rather more than a suspicion that he had a slight longing for his native land; at any rate, it is not difficult to perceive that he enjoys being in Cincinnati. He is succeeded in Basle by Dr. A. V. Elliott, a graduate of the Dental College of the University of Michigan. Dr. E. will doubtless sustain the practice upon which he has entered. May he have success equal to those who have preceded him.

